# CTCF-mediated chromatin loops enclose inducible gene regulatory domains

**DOI:** 10.1186/s12864-016-2516-6

**Published:** 2016-03-22

**Authors:** Martin Oti, Jonas Falck, Martijn A. Huynen, Huiqing Zhou

**Affiliations:** Department of Molecular Developmental Biology, Faculty of Science, Radboud Institute for Molecular Life Sciences, Radboud University, Nijmegen, The Netherlands; Centre for Molecular and Biomolecular Informatics, Radboud Institute for Molecular Life Sciences, Radboud university medical center, Nijmegen, The Netherlands; Department of Human Genetics, Radboud Institute for Molecular Life Sciences, Radboud university medical center, Nijmegen, The Netherlands; Present address: Institute of Biophysics Carlos Chagas Filho (IBCCF), Federal University of Rio de Janeiro (UFRJ), Rio de Janeiro, Brazil

**Keywords:** CTCF, Chromatin looping, Gene regulation, Inducible genes, Transcription factory, Enhancers

## Abstract

**Background:**

The CCTC-binding factor (CTCF) protein is involved in genome organization, including mediating three-dimensional chromatin interactions. Human patient lymphocytes with mutations in a single copy of the CTCF gene have reduced expression of enhancer-associated genes involved in response to stimuli. We hypothesize that CTCF interactions stabilize enhancer-promoter chromatin interaction domains, facilitating increased expression of genes in response to stimuli. Here we systematically investigate this model using computational analyses.

**Results:**

We use CTCF ChIA-PET data from the ENCODE project to show that CTCF-associated chromatin loops have a tendency to enclose regions of enhancer-regulated stimulus responsive genes, insulating them from neighboring regions of constitutively expressed housekeeping genes. To facilitate cell type-specific CTCF loop identification, we develop an algorithm to predict CTCF loops from ChIP-seq data alone by exploiting the CTCF motif directionality in loop anchors. We apply this algorithm to a hundred ENCODE cell line datasets, confirming the universality of our observations as well as identifying a general distinction between primary and immortal cells in loop-enclosed gene content. Finally, we combine the existing evidence to propose a model for the formation of CTCF loops in which partner sites are brought together by chromatin template reeling through stationary RNA polymerases, consistent with the transcription factory hypothesis.

**Conclusions:**

We provide computational evidence that CTCF-mediated chromatin interactions enclose domains of stimulus responsive enhancer-regulated genes, insulating them from nearby housekeeping genes.

**Electronic supplementary material:**

The online version of this article (doi:10.1186/s12864-016-2516-6) contains supplementary material, which is available to authorized users.

## Background

The CCTC-binding factor (CTCF) protein [1] is involved in eukaryotic genome organization, including mediating chromatin interactions [2]. It has functions affecting gene regulation such as enhancer blocking [3] and chromatin domain boundary demarcation [4] where the spread of repressive chromatin is blocked, although the distinction between these two roles has been called into question [5]. It is additionally involved in several more specialized genomic processes including imprinting [6], immune-related genomic recombination and gene expression regulation [7, 8], mammalian X-chromosome inactivation [9], alternative splicing [10] and alternative promoter choice [11]. Interestingly, while it has been described primarily as an insulator protein, it has also been implicated in enhancing gene expression [12], an apparent contradiction. To resolve this contradiction, it has been proposed that CTCF may simply serve to tether distant chromatin sites together, with its different roles depending on the nature of the sites that are brought together and the other DNA-binding proteins involved in the interaction [5].

Genome-wide CTCF-mediated chromatin organization was investigated by Handoko and colleagues in mouse embryonic stem cells [13] with the “chromatin interaction assay using paired-end tags” (ChIA-PET) technique [14]. That study showed that most CTCF interactions link sites on the same chromosome forming loops, which are generally less than 1Mb in length. They also identified an enrichment of the enhancer-associated [15] H3K4me1 (histone 3 lysine 4 monomethylation) chromatin mark within CTCF loops that are <200kb long, constituting the majority of the loops. These within-loop H3K4me1 levels decreased upon knock-down of CTCF. The H3K4me1 chromatin mark is associated with both active enhancers and those poised for future activation, depending on the additional presence or absence respectively of the H3K27ac chromatin mark at the same enhancer [16, 17]. Therefore, this pattern is suggestive of a model in which the <200kb CTCF loops enclose regulatory domains of enhancer-regulated genes. Indeed, a later study using the 5C technique [18] lends support to this model, and proposes a more detailed three-dimensional model in which CTCF interactions demarcate overall regulatory domains within which smaller-scale enhancer-promoter interactions occur.

Recently, three unrelated human patients were identified with disruptive mutations in a single copy of their CTCF gene [19]. These patients showed surprisingly mild phenotypes consisting primarily of mild intellectual disability, reduced head size (microcephaly) and growth retardation. RNA-sequencing data generated from whole blood lymphocytes taken from these patients showed reduced expression levels of enhancer-associated stimulus responsive genes when compared to lymphocytes from healthy controls [19]. This suggests that such genes are incapable of being up-regulated to adequate levels upon induction by stimuli. Patient down-regulated genes were also enriched for enhancer-promoter interactions in RNA Polymerase II ChIA-PET data from K562 leukemia cells [20].

We therefore hypothesized that CTCF interactions may stabilize enhancer-promoter chromatin interactions, facilitating increased transcription levels in response to stimuli. This hypothesis is supported by the recent findings that genome-wide TNF-alpha responsive genes have enhancers and promoters that are already pre-looped prior to their induction [21], and that the TNF-alpha responsive SAMD4A gene is pre-looped head-to-tail by a CTCF-dependent tie before its induction, a configuration that is required for its prompt response to TNF-alpha [22]. Additionally, genes in mouse embryonic stem cells set to be activated later during differentiation are already pre-looped and are associated with chromatin marks for poised enhancers [23]. This enhancer-promoter pre-looping of poised genes has also been observed during *Drosophila* embryogenesis [24] and appears to be a general phenomenon in animal gene regulation.

Here we investigate this hypothesis computationally, seeking to identify whether there is a genome-wide tendency for CTCF loops to enclose stimulus responsive enhancer-regulated genes. We used CTCF ChIA-PET data from human K562 and MCF-7 cells provided by the ENCODE project [25], which are more comprehensive than the mouse CTCF ChIA-PET data analyzed in the previous study. We additionally predicted CTCF loops from genome-wide CTCF binding site data for a hundred cell line datasets provided by the ENCODE project, using an algorithm we developed that exploits the motif directionality in CTCF loop anchors [26, 27]. We find a general tendency for CTCF loops to enclose stimulus responsive genes that are associated with enhancer-based regulation. We also find global differences in loop-enclosed genes between primary and immortal cells, with the former containing more predicted CTCF loop-enclosed genes that are enriched for transcription regulation, cell motility regulation and stem cell differentiation. Finally, we discuss the implications of the motif directionality in CTCF loop anchors, which point to a model of CTCF loop formation involving the reeling of the chromatin template through stationary RNA polymerases, consistent with the transcription factory model of transcription [28].

## Results

### CTCF loops insulate enhancer-regulated stimulus response genes from housekeeping genes

To investigate whether CTCF loops are enriched for enhancers relative to surrounding regions, we investigated the enhancer-associated H3K4me1 (histone 3 lysine 4 monomethylation) as well as other chromatin marks within and around experimentally determined CTCF loops determined using the ChIA-PET technique in K562 myeloid leukemia and MCF-7 breast cancer cell lines. These chromatin mark and ChIA-PET data were all obtained from the ENCODE project [25]. The ChIA-PET CTCF interaction data have been corroborated by the independent Hi-C technique, which does not focus on specific proteins [26]. We focused on CTCF loops less than 200kb in length as these have previously been shown to be associated with active chromatin marks in mouse embryonic stem cells [13]. To ensure we were using reliable ChIA-PET interactions, we filtered them for those containing CTCF binding sites at both anchors, using chromatin immunoprecipitation followed by sequencing (ChIP-seq) datasets also obtained from the ENCODE project [25], and additionally filtered them for those that were supported by at least three ligation products in the ChIA-PET data. Most marks were assessed in the K562 cell line as, being one of the main ENCODE cell lines, it had the largest collection of chromatin mark ChIP-seq datasets.

**Fig. 1:**
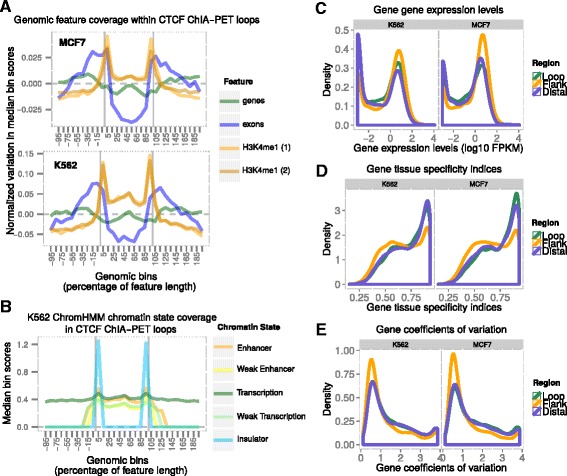
Genomic properties of CTCF loops and flanking regions. **a** Profiles of H3K4me1 histone mark and gene and exon density across genomic regions within and around CTCF ChIA-PET loops in both MCF-7 and K562 cells. The loops and their flanking regions are split into bins each spanning 10 % of the loop length. For each bin, the median feature coverage for all loops is plotted. Profiles were normalized by subtracting the mean of all bins, displaying only the variation pattern across the profile. This was done because mean genomic bin coverage can vary substantially between chromatin marks and other genomic features, separating the profiles along the Y-axis and making pattern comparison more difficult. **b** Profiles of enhancer- and transcription-related chromatin states as defined by the ChromHMM algorithm, within and around CTCF ChIA-PET loops in K562 cells. Processed as above, but without normalizing the means as all the profiles have similar means. Weak transcription differs from the normal transcription state in that it is associated with transcript production but not with any further chromatin marks. **c** Expression level distribution for genes within and flanking CTCF ChIA-PET loops in K562 and MCF-7 cells. Flanking regions are equal in size to the loops they flank. Flanking genes located within neighboring loops are excluded from the set of flanking genes; there is no overlap between loop-enclosed and loop-flanking genes. Expression data are from the same cell lines as the corresponding loop sets. **d** Tissue Specificity Index (TSI, see methods) distribution for genes within and flanking CTCF ChIA-PET loops. Flanking genes are chosen as described above. **e** Coefficient of Variation (CV) distribution for genes within and flanking CTCF ChIA-PET loops. CV (standard deviation/mean) indicates the degree of variability in expression level of a gene across tissues. Flanking genes are chosen as described above

To investigate the loop-flanking regions as well as the loop bodies, each loop was extended on either side to include flanking regions equal in size to the loop itself, after which the extended region was divided into a fixed number of bins each spanning a distance equal to 10 % of the loop length. Average feature density across all loops was calculated per bin, and plotted across the region (Fig. 1a). Consistent with mouse embryonic stem cells [13], we identified an enrichment of the H3K4me1 enhancer mark within CTCF loop bodies relative to flanking regions (Fig. 1a). We did not find enrichment of other histone marks within CTCF loops, except for a slight increase in the H2A.Z histone variant which is also associated with active promoters and enhancers [29] (Additional file 1: Figure S1). However, most histone marks are enriched at CTCF loop anchors (Additional file 1: Figure S1). The enrichment of active enhancer marks within CTCF loops is also detectable using the enhancer states determined by the ENCODE project using the ChromHMM algorithm [30] (Fig. 1b). This algorithm combines different histone marks and other genomic features into a set of states, each defined by a specific combination of features [31]. Chromatin states associated with enhancers, weak enhancers and weak transcription (which is associated with transcript production but no transcription-associated chromatin marks) were strongly enriched within K562 loops relative to flanking regions, while the state associated with normal transcription remained relatively constant across loops and their flanking regions (Fig. 1b). This indicates that while transcription occurs both within and outside the loops, the nature of this transcription differs between the two regions, with enhancer-associated transcription being more prominent within the loops. Unsurprisingly, the insulator state is enriched specifically at the CTCF loop anchors.

We reasoned that enhancer-regulated genes should be located in gene poor regions, as more non-coding DNA within and around the genes would be required for the placement of enhancers [32]. We therefore expected a lower ratio of coding to non-coding DNA within the loops relative to their flanking regions. Indeed, there is a marked reduction in exon density within CTCF loops relative to flanking regions (Fig. 1a). This corresponds to a less pronounced reduction in within-loop gene density, although the centers of the loops are enriched for genes. These results imply that CTCF loops enclose gene poor regions, with a tendency for genes to be centered within the loops. Alternatively, it may reflect a high gene density just outside CTCF loops. The low exon density compared to gene density within the loops relative to the flanking regions indicates a proportionally larger amount of non-coding DNA within the intergenic regions around the loop-enclosed genes, as loop genes are shorter (loop vs flank medians: 13.5kb vs 18.5kb & 13.9kb vs 21.5kb; *p* = 1.2 × 10^−47^ & *p* = 2.4 × 10^−90^ for K562 & MCF-7 cells respectively, Wilcoxon rank sum test) and their intron density is also lower (Additional file 1: Figure S1C).

Gene Ontology [33] analysis showed that genes located within CTCF loops are enriched for response to stimuli (K562 & MCF-7 cells: fold enrichment = 1.2 & 1.2, *p* = 2.5 × 10^−16^ & *p* = 5.7 × 10^−18^ respectively, hypergeometric distribution test). Several stimulus responsive function categories are enriched (particularly immune system and inflammation-related) and, to a lesser extent, developmental and cell differentiation categories (Additional file 2: Table S1). These are biological function categories that are associated with dynamic transcription regulation. Consistent with their biological functions, these loop genes are also enriched for extracellular, plasma membrane and vesicle cellular localizations.

In contrast to genes within the CTCF loops, those in the flanking regions just outside the loops show an expression pattern more similar to housekeeping genes: they are on average more highly expressed than the loop-enclosed genes (Fig. 1c, Table 1), less cell line-specific in their expression pattern (Fig. 1d, Table 1), and have less variation in their expression levels across cell lines (Fig. 1e, Table 1). This is confirmed by the enrichment of genes from a list of human housekeeping genes determined from 16 diverse human tissue types [34] in these loop-flanking regions (K562 & MCF-7 cells: fold enrichments = 1.3 & 1.28 respectively, *p* = 2.91 × 10^−9^ & *p* < 10^−323^ respectively, hypergeometric distribution test). Gene Ontology analysis also shows enrichment for housekeeping biological functions such as RNA processing, primary metabolism, cytoskeletal and cell cycle proteins, with genes localized primarily to the cytoplasm, mitochondria and nucleus (Additional file 2: Table S1). These findings were recently corroborated by another comprehensive study of CTCF ChIA-PET data [35].

**Table 1:** Gene expression pattern differences between ChIA-PET CTCF loop-enclosed, loop-flanking and loop-distal genes

Cell line	Measure	Loops	Flanks	Distal	Flanks/Loops		Flanks/Distal		Loops/Distal	
					FC^a^	*P*-value^b^	FC	*P*-value	FC	*P*-value
K562	Gene Count	4378	4767	6466						
	Median Expression (FPKM)^c^	0.853	1.698	0.091	1.99	5.89e–07	18.66	1.25e–93	9.37	9.74e–56
	Median TSI^d^	0.754	0.6756	0.7565	0.9	5.3e–37	0.89	8.23e–47	1.0	0.27
	Median CV^e^	1.142	0.8357	1.192	0.73	1.72e–36	0.7	1.34e–56	0.96	0.01
MCF7	Gene Count	5469	6490	3847						
	Median Expression (FPKM)	0.467	2.18	0.413	4.67	1.92e–65	5.28	1.07e–90	1.13	9.04e–06
	Median TSI	0.7818	0.6721	0.7549	0.86	1.65e–98	0.89	3.16e–46	1.04	1.19e–05
	Median CV	1.331	0.8214	1.178	0.62	3.92e–100	0.7	9.91e–56	1.13	7.65e–04

^a^FC: Fold Change of the medians

^b^ *P*-value based on Wilcoxon rank sum test with continuity correction

^c^FPKM: Fragments Per Kilobase per Million mapped reads

^d^TSI: Tissue Specificity Index

^e^CV: Coefficient of Variation

Loop-distal genes, which are located neither within loops nor within their flanking regions, generally resemble within-loop genes more than they do loop-flanking genes in their expression patterns in these two cell types, in that they have lower expression levels, higher tissue specificity and greater expression variability across cell types than the loop flanking genes (Fig. 1c–e, Table 1). They further resemble within-loop genes in that they are enriched for cell surface-associated Gene Ontology terms; however these are primarily nervous system-related (Additional file 2: Table S1). It is unknown whether they might be enclosed within CTCF loops in other cell types like neuronal cells.

We have previously reported that genes that were down-regulated in lymphocytes of CTCF mutant human patients are enriched among the within-loop genes for the K562 cell line, after these K562 CTCF loops were filtered for those whose anchors overlap CTCF binding sites in lymphocytes [19]. Here, we additionally detect an enrichment of genes whose expression did not differ between patients and controls, within the CTCF loop-flanking regions of both K562 and MCF7 cells (fold enrichments = 1.13 & 1.18 respectively, *p* = 1.94 × 10^−4^ & *p* = 8.21 × 10^−10^ respectively, hypergeometric distribution test). This is consistent with the housekeeping functions identified for these patient-unaffected genes [19].

### CTCF interactions can be predicted from CTCF ChIP-seq data

**Fig. 2:**
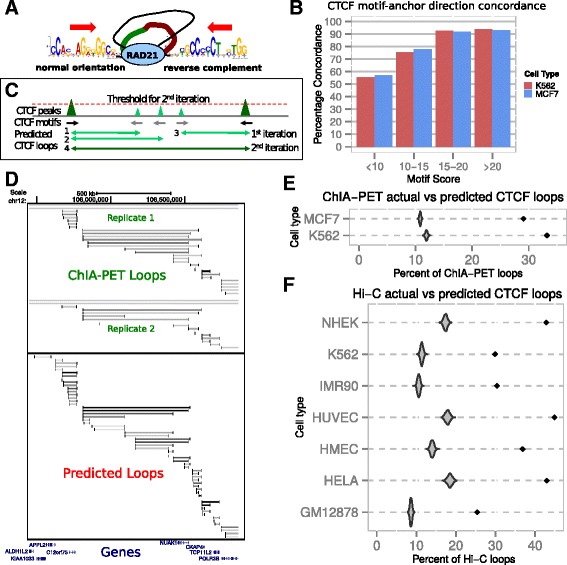
Predicting CTCF loops from ChIP-seq datasets. **a** Model of CTCF loops implied by CTCF motif orientation in loop anchors. RAD21: cohesin subunit. **b** Percentage of motifs for which motif orientation is concordant with loop anchor orientation (as depicted in **a**), for motifs in different score categories in MCF-7 and K562 cells. **c** Schematic illustration of CTCF loop prediction algorithm. Triangles represent CTCF ChIP-seq peaks, with color and size reflecting binding strength (peak scores). Black arrows represent CTCF motifs, with orientation indicated by arrow. Predicted loops are represented by double-headed arrows, with color reflecting predicted loop strength. Briefly, the loop prediction algorithm scans the genome from left to right connecting start anchors to end anchors based on motif orientation (1^st^ iteration, light green horizontal arrows). New loop sets are begun when new start anchors are encountered beyond end anchors (loop 3). Subsequently, weaker peaks (light green triangles) are filtered out and the genome is rescanned, identifying stronger, longer loops (loop 4, dark green arrow). This process is repeated with progressively higher peak thresholds up to a maximum threshold. **d** Example of CTCF ChIP-seq peak-based predicted CTCF loops (below) compared to experimentally determined CTCF ChIA-PET loops (above, data from two replicate experiments shown) in MCF-7 cells. Darker lines indicate stronger (predicted) loops. **e** Comparison of percentage of correctly predicted ChIA-PET loops with 1000 random control datasets in which motif orientation within ChIP-seq peaks is randomly permuted, for K562 and MCF-7 cell lines. Grey density plots depict the distributions of the random control percentages. Black diamonds indicate the actual percentages. **f** Comparison of percentage of correctly predicted Hi-C loops with 1000 random control datasets in which motif orientation within ChIP-seq peaks is randomly permuted, for 7 cell lines

In order to investigate CTCF loop-enclosed genes in a broad array of cell lines, we developed an algorithm to predict CTCF loops from CTCF ChIP-seq datasets. These datasets contain genome-wide CTCF DNA binding sites and are available for many cell lines from the ENCODE project [25]. Our algorithm exploits the recently reported finding that CTCF binding site motifs are oriented towards the loop body [26]. Using ENCODE CTCF ChIA-PET data from the K562 and MCF-7 cell lines, we first confirmed that the 3′ end of the CTCF motif (Jaspar database [36]) is oriented towards the loop body (Fig. 2a) in over 70 % of all anchors (K562: 27946/38452 anchor motifs (73 %), MCF-7: 48653/67584 anchor motifs (72 %), *p* < 10^−323^ for both K562 and MCF-7 cell lines, chi-square test). We further observed that the concordance between motif orientation and interaction direction correlates with motif score; it increases with the similarity of the motif to the canonical CTCF motif, reaching almost 95 % concordance for the highest-scoring motifs (Fig. 2b). Concordant CTCF motif scores follow a bimodal distribution with an enrichment of high-scoring motifs as well as a subset of lower-scoring motifs, while discordant motifs follow a unimodal distribution with primarily low-scoring motifs (Additional file 1: Figure S2A).

Based on this motif orientation preference, we developed a computational algorithm for predicting CTCF interactions from ChIP-seq data (Fig. 2c). For a given ChIP-seq dataset, we scan the CTCF motifs located in the CTCF binding sites (ChIP-seq peaks), opening CTCF loops each time a parallel motif is encountered and closing all currently open loops for each anti-parallel motif that is encountered. As this simple scanning approach cannot identify nested CTCF loops which occur extensively in the CTCF ChIA-PET data, we iteratively rescan the genome after removing the lowest-scoring ChIP-seq peaks from the dataset, using successively more stringent cut-offs. Given that stronger peaks are more likely to be involved in experimentally determined interactions [13], removing weaker peaks should allow us to skip over CTCF binding sites that do not interact robustly in the cell. This allows us to identify larger outer loops that enclose smaller inner loops, provided the outer loops are anchored by stronger CTCF binding sites than the inner loops (Fig. 2c). When predicted loops at different peak thresholds are combined, this approach effectively recovers nested ChIA-PET loop structures (Fig. 2d). We find the highest recovery rate of ChIA-PET loops occurs when excluding the weakest 30–50 % of CTCF ChIP-seq peaks, although the success rate varies between different CTCF ChIP-seq data sets even for the same cell type (Additional file 1: Figure 2B). Nevertheless, prediction performance is much better than random expectation for both K562 and MCF-7 cell types (~30 % vs ~12 %, *p* < 10^−323^ for both cell types, *p*-value based on normal distribution fitted to random scores) (Fig. 2e). Independent validation using experimentally determined CTCF-associated loops based on the Hi-C technique [26] gave consistent results with the ChIA-PET loop validation (*p* < 10^−323^ for all cell types, *p*-value based on normal distribution fitted to random scores) (Fig. 2f). It should be noted that these overlap estimates are probably lower bounds, as the ChIA-PET technique is likely to miss interactions as evidenced by the moderate reproducibility of loops between replicates (~11800 shared loops between replicates containing 47234 and 18181 loops respectively), while the Hi-C loops are biased toward longer loops due to the sequencing depth-limited genomic resolution of the technique [26] (Additional file 1: Figure S2C). In addition, the overall domain architecture can be detected from the predicted loops even when many of the individual ChIA-PET loops within the region are undetected (Fig. 2d). Furthermore, predicted loops involving stronger CTCF peaks are more likely to reflect experimentally determined loops than those linking weaker peaks (Additional file 1: Figure S2D), consistent with the observation that stronger CTCF peaks are more likely to be involved in ChIA-PET interactions in mouse embryonic stem cells than weaker CTCF peaks [13]. When all peaks are used for loop predictions, the majority of the genome falls within predicted CTCF loops in most cell lines (Additional file 3: Table S2). Predicted loops involving weak CTCF peaks are therefore less likely to be biologically relevant than those linking stronger binding sites.

In order to obtain subsets of high-confidence predicted loops, we filtered the top-scoring 5 % of predicted loops for each dataset. As these high-scoring predicted loops also tend to be the longer loops (Fig. 2c), they reflect the larger CTCF-demarcated domains and generally enclose 25–30 % of all genes predicted to lie within CTCF loops per cell line (Additional file 3: Table S2). These stringent loop subsets were used for further comparison of the genome-wide CTCF loop-enclosed gene architecture across the ENCODE cell lines.

### Enrichment of stimulus response genes within CTCF loops is universal across cell lines

**Fig. 3:**
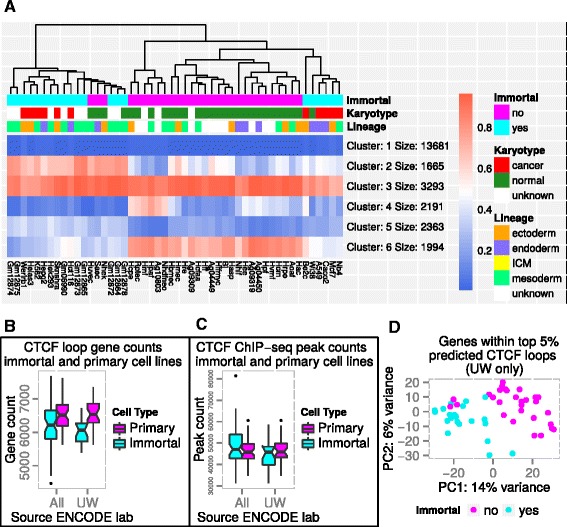
Gene content similarities and differences between ENCODE cell lines for predicted CTCF loops. **a** Heatmap showing similarity of predicted CTCF loop-enclosed gene content across the University of Washington ENCODE cell lines. Genes are clustered into six clusters using K-means clustering. Cell lines are clustered hierarchically based on the K-means cluster patterns, using the average linkage algorithm and Euclidean distances. Heatmap colors indicate the proportion of genes from that cluster falling within CTCF loops in that cell line. **b** Predicted CTCF loop gene count distributions for primary and immortal cells, for all ENCODE datasets and for the subset from the University of Washington (UW). Notches extend 1.58*IQR/sqrt(n) from the medians and roughly correspond to their 95% confidence intervals (IQR = inter-quartile range). **c** CTCF ChIP-seq peak count distributions for primary and immortal cells (all ENCODE datasets and UW-only datasets). **d** Principal Components Analysis (PCA) of cell lines based on their predicted CTCF loop-enclosed gene content (PC1 vs PC2 plotted)

We applied our CTCF loop prediction algorithm to 100 CTCF ChIP-seq datasets generated by the ENCODE consortium [25], in order to investigate the generality of the enrichment of stimulus response genes within CTCF-enclosed chromatin domains (Additional file 3: Table S2 and https://zenodo.org/record/29423). Due to the variability in ChIP-seq datasets and correspondingly in loop prediction between different labs even for the same cell line (Additional file 1: Figure 2B), we restricted our analysis to the 50 cell line datasets generated by the University of Washington (UW) [25]. Using K-means clustering, we clustered the genes into six clusters based on their pattern of loop membership across cell lines (Fig. 3a). Similar results are obtained with different choices of cluster number (Additional file 1: Figure S3). While the largest cluster (cluster 1; 13681 genes) contains genes absent from strong predicted CTCF loops in virtually all cell lines, the next largest (cluster 3; 3293 genes) contains genes predicted to lie within strong CTCF loops in the majority of cell lines. Consistent with the genes located within ChIA-PET loops in K562 and MCF-7 cells, this latter cluster is enriched for genes involved in the regulation of cellular responses to external signals (Additional file 4: Table S3).

Strikingly, hierarchical clustering of the cell lines based on their cluster similarity profiles reveals a general segregation of primary and immortal cells (Fig. 3a). Cluster 2 (1665 genes) contains genes that are loop-enclosed predominantly within immortal cells, while cluster 6 (1994 genes) contains genes that generally loop-enclosed in primary cells but not in immortal cells. Primary cells also generally contain more predicted loop-enclosed genes than the immortal cells (median 6533 vs 6063 genes respectively for UW samples, *p* = 3.47 × 10^−5^, Welch two sample *T*-test), an effect that is still observable even when datasets from multiple ENCODE labs are pooled (Fig. 3b). This is despite the fact that the overall number of CTCF binding sites does not differ significantly between these two classes (median 45910 vs 45711 peaks respectively for UW samples, *p* = 0.28, Welch two sample *T*-test) (Fig. 3c, and ref. [37]), and that CTCF gene expression is actually higher in immortal than in primary cells [37]. This segregation of primary and immortal cells is also observable using principal components analysis (Fig. 3d) where it corresponds to the main axis of variation. It is consistent with the previously reported DNA methylation-dependent differences in CTCF binding patterns between these two classes of cell types [37], in which a small subset of CTCF binding sites is silenced by DNA methylation in immortal cells [38]. Interestingly, and consistent with their more differentiated nature, gene ontology [33] analysis shows that genes within primary cell-specific predicted CTCF loops are enriched for transcription regulation, stem cell differentiation and the regulation of cell motility and migration (Additional file 4: Table S3). In contrast, genes located within putative CTCF loops in immortal cells but not in primary cells show a slight enrichment for regulation of (Rho) GTPase activity, lipid and glucan metabolic processes, and mitotic cell cycle – consistent with their proliferation phenotype (Additional file 4: Table S3). Apart from this separation into primary and immortal cell types, no clear clustering by cell type is observable (Fig. 3a). Somewhat surprisingly, three primary cell types (HUVEC, NHEK & SAEC) cluster with the immortal cell lines (Fig. 3a).

A common theme to the functions associated with gene clusters enriched for CTCF loop-enclosed genes in multiple cell types (clusters 2,3,4 & 6) is the preponderance of regulatory processes. In contrast, genes that are rarely or never located within putative CTCF loops (clusters 1 & 4) are enriched for housekeeping processes and for genes that are involved in specialized and highly cell type-specific biological processes, such as olfactory receptors (Additional file 4: Table S3). This is consistent with the observation that genes with complex regulatory requirements have intermediate levels of tissue-specificity in their expression patterns [32], and with CTCF playing a role in the genomic organization of genes requiring dynamic regulation and rapid changes in expression levels.

## Discussion

Based on the pattern of affected genes in lymphocytes of human patients with mutations in a single copy of their CTCF gene [19], we hypothesized that a major role of CTCF-mediated chromatin interactions may be to stabilize the three-dimensional enhancer-promoter chromatin conformations of inducible genes. Here our data show that stimulus response genes are enriched within the CTCF loops across a wide variety of cell types, providing evidence to support this model.

**Fig. 4:**
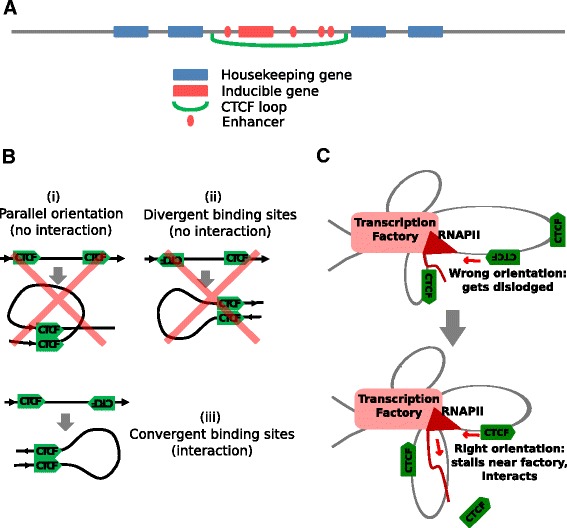
Models of CTCF loop formation and genomic organization. **a** Model of CTCF loop-enclosed genomic domain. CTCF loops enclose domains of enhancer-regulated stimulus-responsive genes, separating them from domains of constitutively expressed housekeeping genes. **b** The strong preference shown by CTCF binding sites for interacting with partner sites on their 3′ side that have an opposite orientation cannot be explained by a spatial diffusion model in which partner sites encounter each other through diffusion in three-dimensional nuclear space. Such a mechanism would not be able to distinguish between the three depicted scenarios (upstream vs downstream location of partner sites and parallel vs anti-parallel orientation of partner sites). The strong observed preference for scenario (iii) implies chromatin template-mediated contact between the partner sites. **c** Mechanistic model for the formation of interactions between CTCF-bound genomic sites. Relatively stationary RNAP2 molecules in transcription factories reel in the chromatin template, bringing distal CTCF binding sites towards the factory. Bound CTCF molecules presenting their C-terminus to the RNAP2 polymerase interact with and are efficiently dislodged by the polymerase. Those presenting their N-terminus stall the polymerase, giving them time to interact with other factory-tethered CTCF molecules

Interestingly, CTCF-enclosed chromatin domains are enriched for H3K4me1 chromatin mark which marks both active and poised enhancers [16, 17], but they are not as enriched for the H3K27ac mark which is specific to active enhancers and promoters [16, 17] (Fig. 1a, Additional file 1: Figure S1). Given CTCF’s previously described role in poising inactive inducible genes for activation [22], this suggests that they are associated with the poising of inducible genes for transcription whether or not they are currently active. In support of this, the loop-enclosed genes tend to have a greater expression variation within cell types and increased expression variability between cell types relative to loop-flanking genes (Fig. 1c–e; see also ref [35]). In contrast, these flanking genes are generally widely and highly expressed across cell types (Fig. 1c–e; see also ref [35]), but are not associated with enhancer marks (Fig. 1b). The gene functions enriched within loops are those associated with inducible genes, while those enriched among flanking genes are associated with constitutive housekeeping processes. This leads to a model in which CTCF loops enclose regulatory domains of dynamically expressed inducible enhancer-regulated genes, insulating them from nearby domains of constitutively expressed housekeeping genes (Fig. 4a). This model is consistent with Hi-C data that show that flanking regions of topologically associating domains are enriched for housekeeping genes [39], and that domain boundaries are demarcated by CTCF loops [26], and has been corroborated in another recent study [35].

These findings are also consistent with the genes affected in CTCF patient lymphocytes, where highly expressed genes involved in response to stimuli were expressed at lower levels in patients than in controls [19]; a pattern which suggests that those patient genes were not able to be sufficiently induced in response to stimuli. In contrast to these patient lymphocyte findings, a recent study in which CTCF was knocked down in HEK293T cells found very little effect on global gene expression [40]. However, if CTCF loss primarily affects gene induction, such an effect would only be detected if the cells were additionally provided with transcription-inducing stimuli. Notably, in the CTCF patient lymphocytes, the affected genes were primarily involved in response to wounding, inflammation and bacterial defense [19], all processes that can be expected to be activated in lymphocytes in blood drawn from human individuals.

The enclosure of the regulatory domains of stimulus response genes is consistent with CTCF’s bilaterian multicellular animal phylogenetic distribution [41], as organisms in this clade have complex developmental processes that make extensive use of local environmental signaling cues within the embryo, such as morphogen gradients and cell surface molecular markers, to direct cell differentiation, migration, adhesion, proliferation and apoptosis during development [42]. Diversification of cell surface proteins and nuclear transcription factors, which expand a cell’s repertoire of responses to environmental signals, is characteristic for the evolution of multicellular animal development [43]. Our findings therefore support CTCF’s proposed role as a key facilitator of the evolution of complex multicellular animals [41].

Mechanistically, the poising of inducible genes by CTCF may involve the stabilization of the chromatin interaction hubs that maintain the spatial co-localization of enhancers and promoters [18, 26, 44, 45]. Indeed, a CTCF interaction has been described to stabilize a head-to-tail looping interaction of the inducible SAMD4 gene, an interaction that is required for prompt response of the gene to an inducing stimulus [22]. Interestingly, the convergent linear orientation of interacting DNA-bound CTCF molecules [26] (Fig. 2a) implies that such enhancer-promoter chromatin interaction hubs are not only required for transcription, but are also formed by transcription. This is because such an orientation preference is best explained by a transcription model in which static fixed motor molecules reel a mobile chromatin template through themselves (Fig. 4b and c). Within the nucleus, motif orientation along the chromatin strand cannot be reliably determined if CTCF binding sites encountered each other through spatial diffusion, as interacting sites would not be able to distinguish between parallel and anti-parallel orientations of their chromatin strands (Fig. 4b; see also ref. [46, 47]). Similarly, oppositely oriented binding sites would not be able to determine whether they are located 5′ or 3′ to each other without information on their linear chromatin positions relative to each other (Fig. 4b). Given that CTCF binding sites primarily interact with partner sites that are oppositely oriented, and given that these sites generally lie on their 3′ side, there must be linear communication between them along the chromatin strand (see also ref. [46, 47]). Additionally, as linearly distant partner sites need to be brought together in close spatial proximity in the nucleus in order to interact with each other, this linear communication can be explained by chromatin template reeling by a motor protein that pulls the partner sites towards each other (Fig. 4c). The most obvious candidate for such a reeling motor is RNA polymerase II (RNAPII). It has been proposed that during RNAPII transcription relatively static polymerases reel the mobile chromatin template through them as they transcribe DNA [48]; a central tenet in the “transcription factory” model of transcription [28]. CTCF interacts with RNAPII via its C-terminal domain [49], although the loop body is located on the 3′ side of the motif and therefore on the N-terminal side of the bound protein [44, 50] (Fig. 2a). DNA-bound CTCF molecules have also been reported to stall transcribing RNAPII polymerases [51].

These observations allow us to propose a mechanistic model for CTCF binding site interaction formation, in which reeling polymerases in transcription factories stall when they encounter bound CTCF molecules that they cannot interact with (Fig. 4c). The more strongly the CTCF molecule is bound the longer the polymerase is stalled, increasing the opportunity for interaction with spatially proximal CTCF binding sites also localized to the transcription factory – explaining the quantitative relationship between binding strength and orientation preference (Fig. 2b). Should the reeling polymerase encounter a bound CTCF molecule presenting its C-terminal domain however, it would be able to interact with and efficiently dislodge the bound CTCF molecule (Fig. 4c). In this model, transcription would only be required for loop formation and not loop maintenance. This loop formation-associated transcription through entire gene regulatory domains may be related to the “weak transcription” chromatin state which is specifically enriched within CTCF loops (Fig. 1b). It is important to note that such domain-wide transcription is unlikely to produce functional mRNAs and therefore does not conflict with the observation that enhancer-promoter chromatin interactions can be formed and maintained in the absence of active mRNA production [21, 23]. Once formed, the CTCF interactions can then be fortified through the recruitment of cohesin ties [52, 53]. This is supported by the obsevations that the majority of CTCF binding sites are co-bound by cohesin and that CTCF binding is required for cohesin recruitment to these sites [54]. Our model is similar to the extrusion model recently proposed by Nichols and Corces [46] as well as Lieberman‐Aidez and colleagues [47], but proposes a central role for transcription as the motor behind CTCF loop formation. Nevertheless the two models are not mutually exclusive.

It is reasonable to assume that not all interacting CTCF binding sites are brought together by chromatin template reeling as inter-chromosomal and very long range cis-interactions also occur [13]. While some of these long-range interactions might be artifacts of the ChIA-PET technique, inter-chromosomal interactions identified using ChIA-PET have been experimentally validated [20, 23], so many are likely to be genuine. Therefore, chromatin template reeling is unlikely to be an absolute requirement for the establishment of all CTCF interactions. Actively transcribed sites may also co-localize in the nucleus through spatial clustering of active genes in transcription factories [55], even without RNAPII reeling as a mechanism to bring them together. Still, the preponderance of specifically located and oriented cis-linked interaction partners, especially for the binding sites with stronger motifs (Fig. 2b and ref. [26]), suggests that chromatin template reeling is the main mechanism by which CTCF binding sites are brought into proximity with their interaction partners. Importantly, this model suggests that transcription shapes the formation of the CTCF interactome.

It should be noted that the gene functional category enrichments for the CTCF loop-enclosed and loop-flanking genes are relatively modest, generally below two-fold (Additional file 2: Tables S1 and Additional file 4: Table S3), and therefore probably only reflect the role of a subset of CTCF loops. The majority of CTCF loops may simply be involved in the structural organization of the chromatin fiber. Nevertheless, the H3K4me1 poised/active enhancer mark is the only one that is clearly enriched within CTCF loops when they are viewed at a global scale (Additional file 1: Figure S1), and the enrichment of genes involved in regulatory and stimulus responsive processes within CTCF loops is similarly clear and consistent across cell types (Fig. 3a, Additional file 2: Tables S1 and Additional file 4: Table S3). Where they do differ between cell types (primary versus immortal), the putative loop-enclosed genes reflect regulatory and environmental interaction-related cellular processes that are relevant to the cell types in which these genes are loop-enclosed (Fig. 3a, Additional file 4: Table S3). Additionally, in lymphocytes of patients with reduced levels of functional CTCF molecules, actively transcribed stimulus inducible genes are clearly reduced [19]. These patients also have mild phenotypes, suggesting that most functions of CTCF are not very dosage-sensitive. Taken together, these observations indicate that its role in poising inducible genes for transcriptional up-regulation, whether in response to environmental signals [22] or in the course of normal development and cell differentiation [56], is one of the most important general roles of CTCF in the nucleus – at least from a quantitative perspective.

## Conclusions

A major role of CTCF-mediated chromatin loops, particularly those that are less than 200kb long, appears to be to enclose enhancer-regulated gene domains, particularly those involved in responding to stimuli. This looping may facilitate rapid changes in transcription rate by stabilizing pre-formed enhancer-promoter chromatin hubs that can readily be converted into, or recruited to, active transcription factories. It may also permit such genes to be controlled independently from neighboring constitutively expressed housekeeping genes. CTCF-mediated chromatin loops can be predicted from ChIP-seq data due to the CTCF binding orientation preference at interacting loop anchors. This orientation preference suggests that these loops may be formed by relatively stationary RNAPII molecules reeling in the chromatin template, thereby bringing together distant CTCF genomic binding sites into close spatial proximity in the nucleus.

## Methods

### Data sets

All analyses were done using the hg19/GRCh37 assembly of the human genome. CTCF ChIA-PET loop and CTCF ChIP-seq peak files for the K562 and MCF-7 cell lines were taken from the ENCODE project [25], downloaded from the ENCODE data collection center (DCC) of the UCSC genome browser website [57]. The final sets of predicted CTCF loops for these cell lines, used for validation against the ChIA-PET loops, were generated by merging the ChIP-seq peak datasets per cell line, retaining the maximum score per peak where they overlapped. The CTCF ChIA-PET interactions were filtered for intra-chromosomal loops that overlapped CTCF ChIP-seq peaks at both anchors in the same cell line. Peaks from replicate ChIP-seq experiments were merged per cell line. Histone mark ChIP-seq mapped read BAM files for both cell lines were also downloaded from the ENCODE DCC at the UCSC. As the ENCODE ChIP-seq datasets did not include the H3K4me1 mark for the MCF-7 cell line, the BAM files for this cell line (from two replicate ChIP-seq experiments) were obtained from reference [58]. All available ENCODE CTCF ChIP-seq peak files for the different cell lines and from different labs were downloaded from the UCSC ENCODE DCC. Only the “AWG” versions of these ChIP-seq datasets were used for cell line-specific CTCF loop prediction, as these were all generated using a standardized processing pipeline by the ENCODE consortium Analysis Working Group [25]. Genomic locations of genes and exons were downloaded from the ENSEMBL database version 75 (human genome assembly GRCh37) [59] using the BioMart web interface [60]. Gene Ontology terms [33] and their human gene associations were downloaded from the FTP website of the Gene Ontology Consortium (ftp://ftp.geneontology.org/) on April 9 2015. ChromHMM chromatin state datasets were downloaded from the UCSC ENCODE DCC, with states defined by the ENCODE project [31]. RNA-seq expression data (FPKM values) for polyadenylated whole cell gene transcripts, generated at the CSHL lab using the GENCODE transcriptome [61] version 7, were downloaded from the UCSC ENCODE DCC for 15 cell lines (Additional file 3: Table S2).

### Analysis tools and scripts

All analyses were performed using custom-written Python, R and shell scripts (https://zenodo.org/record/29423), in addition to third party Linux command line tools. In particular, the BEDTools suite [62] (versions 2.16.2 to 2.22.1) was used extensively for genomic interval processing. Statistical analyses were carried out using the R statistical program (R core team (2015) R: A language and environment for statistical computing. R foundation for Statistical Computing. Vienna, Austria. http://www.R-project.org/). Plotting was done with R using the ggplot2 [63] and pheatmap (http://cran.r-project.org/web/packages/pheatmap/) packages. Gene Ontology term enrichment was performed using the goseq R package [64] from the Bioconductor project [65].

### Genomic profiles around CTCF loops

The reproducibility between the MCF-7 ChIA-PET replicates is moderate (~11800 shared loops between replicates containing 47234 and 18181 loops respectively). Therefore we would expect clearer results when restricting our analysis to better-supported loops. Due to the large difference in total number between the replicated and the presence of well-supported loops (in terms of ligation products) in only a single of the two replicates, we decided to use a more stringent cut-off of 3 ligation products (as opposed to the default 2) for loop filtering rather than restricting ourselves to loops present in both replicates. We indeed obtained stronger over-representations and more significant *p*-values with the more stringent loop set, although similar results were also obtained with the full loop set (data not shown).

Genomic profile generation around CTCF loops was performed using a similar approach to that used by Handoko et al. [13]. Each loop was extended by an equal-sized region on either flank. Loops that were extended past a chromosome end were filtered out. The extended loop regions were split into 30 bins, each equal to 10 % of the original loop length. For each feature type (ChIP-seq reads, exons, genes), features were overlapped with bins for each CTCF loop, and feature counts per bin were normalized into FPKM values (Features Per Kilobase per Million features) to account for differing bin sizes between loops and differing total feature counts between feature types. Subsequently, for each of the 30 bins the median FPKM score across all loops was determined. This resulted in a single score profile per dataset. These score profiles were mean-normalized to zero by subtracting the mean score of the 30 bins for each profile, centering all profiles on zero. This was done in order to facilitate the comparison of histone mark profiles with different median coverage levels in the same plot, given that some marks have higher average densities than others. However this mean-normalization was not performed for the ChromHMM plot in Fig. 1b, as the signal levels were comparable for all profiles. Consequently these are absolute median FPKM profiles and are not zero-centered. Note that as median feature FPKMs are used per bin, a score greater than zero is generated only when the feature occurs in that bin in at least half the surveyed loops. Variance was not normalized in these profiles, to retain the differences between features in the relative scale of the variation. Genomic profile generation was carried out using a custom R script (https://zenodo.org/record/29423) which in turn uses the BEDTools programs makewindows (for splitting of extended loop regions into bins), intersect (for counting features in window bins) and bamtobed (for reading from ChIP-seq BAM files). For the K562 cell line the single CTCF ChIA-PET dataset was used for profile generation while for the MCF-7 cell line, which has two replicate CTCF ChIA-PET datasets, the larger dataset (replicate 1) was used. Both datasets were filtered for loops supported by at least three ligation products.

### Gene expression patterns within and adjacent to CTCF loops

Genes were classified as being within-loop genes if they overlapped a CTCF ChIA-PET loop, and as loop-flanking genes if they were located within loop-flanking regions of equal size to the loop. Genes that fell into both categories because they flanked some loops but overlapped others were subtracted from the set of loop-flanking genes. Other genes not falling within either of these two categories were classified as loop-distal genes.

Gene expression patterns were determined using a subset of ENCODE RNA-seq datasets, limited to the GENCODE version 7 whole cell PolyA+ transcript RNA-seq datasets from the Cold Spring Harbor Laboratory (CSHL) for consistency reasons. This resulted in expression data for 15 cell types including K562 and MCF-7 (Additional file 3: Table S2). The ENCODE-computed FPKM values were used, and the mean of the two replicates was taken per gene.

The Tissue Specificity Index (TSI) was taken from Yanai et al. [66] and determines to what extent a gene is expressed at a much higher level in one or a few tissues relative to the rest. It is given by:

$$ \mathrm{T}\mathrm{S}\mathrm{I} = {\sum_{\mathrm{i}}}^{\mathrm{n}}\left(1 - {\mathrm{E}}_{\mathrm{i}}/{\mathrm{E}}_{\max}\right)/\left(\mathrm{n}-1\right) $$

where E_i_ is the expression level in tissue *i*, E_max_ is the maximal expression level in any tissue and n is the number of tissues or cell types.

The Coefficient of Variation (CV) is a normalized measure of the variance in expression level across the datasets, and is given by:

$$ \mathrm{C}\mathrm{V} = \mathrm{S}\mathrm{D}\left(\mathrm{E}\right)\ /\ \mathrm{mean}\left(\mathrm{E}\right) $$

where E is the set of expression levels in all tissues, and SD and mean indicate the standard deviation and the mean of the gene expression levels across the cell types, respectively.

The list of human housekeeping genes were taken from Eisenberg & Levanon 2013 [34].

### Motif-anchor concordance levels

To determine the concordance between CTCF motif and ChIA-PET loop anchor orientations, CTCF motifs were intersected with ChIA-PET anchors using BEDTools intersect for both the K562 and the MCF-7 (replicate 1 dataset) cell lines. As anchors for multiple different ChIA-PET loops can localize to the same CTCF binding site, the total number of anchors in each orientation was tallied for each motif, as well as the maximum ChIA-PET loop score for each orientation. For the ChIA-PET loop score, the number of ligation products supporting the reported ChIA-PET loop was used. Motifs overlapped by ChIA-PET anchors in both orientations were classified as bi-directional. For comparing concordance levels for different motif score bins, bi-directional anchor motifs were classified as discordant if one or more discordant loops were supported by more ligation products than all concordant interactions (i.e. if one or more discordant loops were stronger than all concordant loops), and otherwise as concordant.

### CTCF loop prediction algorithm

The FIMO tool [67] from the MEME suite [68] (version 4.8.1) was used to scan genome-wide for CTCF motifs in the human genome (assembly hg19) (Additional file 3: Table S2). Default settings were used, except for a conservative *p*-value threshold of 2.5 × 10^−4^ which was chosen due to the *p*-value distribution acquiring a strongly periodic pattern above this threshold (Additional file 1: Figure S4), suggesting primarily artifactual motifs. The CTCF motif position weight matrix used for the scanning was taken from the JASPAR database [36], from the JASPAR CORE vertebrates motif collection of 2009 (motif ID: MA0139.1). For each ChIP-seq peak dataset, the genome-wide motifs were overlapped with the peaks to obtain the subset of peak motifs for that dataset. Peak-contained motifs were considered as anchors for loop prediction. Loop anchor scores were computed from both peak and motif scores by multiplying the ChIP-seq peak scores provided by the ENCODE consortium with the motif scores assigned by FIMO, weighting the peak scores tenfold more than the motif scores:

$$ \mathrm{anchor}\_\mathrm{score} = \mathrm{peak}\_\mathrm{score}\ *\ \left(\mathrm{motif}\_\mathrm{score}/10\right) $$

This was done because CTCF binding strength can vary between cell types at the same binding sites, in a DNA methylation-dependent manner [37]. Consequently, the experimentally determined binding strength in each cell line was weighted more heavily than the static motif score. Loop scores were calculated as the geometric mean of the anchor scores at both anchors.

In this study the prediction algorithm iterates over thresholds defined using peak proportions rather than peak scores. However, the Python script created to implement the algorithm is capable of using either proportions or scores.

The basic loop prediction algorithm used in this study is as follows:

1. For all chromosomes:
  - Scan through all CTCF peak-enclosed motifs.
  - If it is a plus strand motif and there are no current end anchors, add it to the list of current start anchors.
  - If it is a plus strand motif and there are one or more current end anchors, match all current plus strand motifs (start anchors) to all current minus strand motifs (end anchors) to create a set of predicted loops. Score all newly predicted loops with the geometric mean of their two anchor scores (less outlier sensitive than arithmetic mean). Then initiate a new list of current start anchors with the present one.
  - If it is a minus strand motif, add it to the list of current end anchors.
  - If the end of the chromosome is reached, match all current plus strand motifs (start anchors) to all current minus strand motifs (end anchors).
2. Filter out lowest scoring 10 % ChIP-seq peaks and repeat step 1 above. Merge the new predicted loops with the already existing predicted loops.
3. Repeat step 2 above with successively larger subsets of the lowest scoring peaks being filtered out, in increments of 10 % of the total number of ChIP-seq peaks, until the final threshold where 60 % lowest scoring peaks have been filtered out.

The genome is only scanned in the forward direction, as with this algorithm the reverse scan gives identical loop predictions and is therefore redundant. A custom Python script implementing this algorithm is provided in the Additional files (Additional file 5).

### CTCF loop prediction validation

Predicted CTCF loops were validated against ENCODE ChIA-PET loops for the K562 and MCF-7 cell lines, and against the Hi-C loops from Rao et al. [26] for the seven cell lines provided therein. ENCODE ChIP-seq datasets were merged per cell line using the BEDTools “merge” tool, and merged peaks were assigned the maximum peak score from the contributing datasets. ChIA-PET loops were filtered for those supported by at least 3 ligation products. Both MCF-7 ChIA-PET replicate datasets were merged before validation. CTCF motifs that served as anchors in predicted loops were extended by 100 bp in either direction before overlapping with the experimental loop anchors. Only predicted loops that overlapped experimentally determined loops at both anchors were considered validated. Predicted versus experimental loop overlap evaluation was done using a custom Python script (https://zenodo.org/record/29423), which in turn relies on BEDTools intersect.

Randomization controls for the predicted CTCF loop datasets were generated by randomly permuting the motif orientations of the peak-contained CTCF motifs. Everything else remained the same, including peak and motif locations and strengths, and the total numbers of motifs in each orientation. K562 and MCF-7 dataset randomizations that were validated against the ChIA-PET loops were performed using the same merged ChIP-seq peak datasets used for loop prediction in these cell lines. Randomizations for the datasets validated against the Hi-C loops were based on the AWG ChIP-seq peak datasets, which were also used for the actual loop predictions.

### ENCODE ChIP-seq dataset comparison

CTCF loops were predicted for the 100 ENCODE AWG uniformly processed ChIP-seq datasets. Predictions were performed using a final peak threshold of 60 % and an interval size of 10 %. For each dataset the top 5 % of predicted CTCF loops less than 1Mb long were used for further analyses. Genes contained within these loops were determined using BEDTools intersect. Five out of the 100 datasets with abnormally low numbers of loop-enclosed genes, defined as greater than 2 standard deviations from the mean number of predicted CTCF loop-enclosed genes per dataset, were excluded as being outliers. The 50 University of Washington (UW) cell line datasets from the remaining 95 ChIP-seq datasets were used for further analyses.

The pheatmap R package was used for the K-means clustering of genes based on their pattern of predicted loop containment across the 50 UW ENCODE cell lines, as well as the hierarchical clustering of these cell lines based on their K-means cluster score profiles. The K-means cluster scores indicate the proportion of genes in that cluster that lie within predicted CTCF loops in that cell line.

Classification of ENCODE cell line datasets according to different properties such as lab of origin, cell lineage, karyotype and immortality was done based on the ENCODE annotation available from the UCSC ENCODE Data Collection Center website (http://genome.ucsc.edu/ENCODE/cellTypes.html).

## Availability of supporting data

Additional data files have been submitted to the Zenodo website and are available online at https://zenodo.org/record/29423 [69].

## Additional files

Additional file 1: Figure S1. Profiles of histone marks and gene-related features across genomic regions within and around CTCF ChIA-PET loops in K562 cells. The loops and their flanking regions are split into bins each spanning 10 % of the loop length. For each bin, the median feature coverage for all loops is plotted. Profiles were normalized by subtracting the mean of all 30 bins, displaying only the variation pattern across the profile. This was done because mean genomic bin coverage can vary substantially between chromatin marks and other genomic features, separating the profiles along the Y-axis and making pattern comparison more difficult. A) Loops shorter than 200kb in length. B) Loops between 200kb and 1Mb in length. (A & B) Top panels: regulatory element-associated histone marks and variants. Middle panels: Repression-associated histone marks. Bottom panels: Transcribed region-associated histone marks. C) Gene-related features for K562 (top) and MCF-7 (bottom) cell lines. (Note that as median densities are plotted per bin, when the proportion of loops with a given feature drops below half the median jumps to zero as in the peripheral bins of the MCF-7 DHS profile. Also note that the strong DHS enrichment at loop anchors compresses the scale of the other profiles.). **Figure S2**. A) Motif score distributions for exclusively concordant, exclusively discordant and bi-directional loop anchors in both MCF-7 and K562 cells. Concordant and bi-directional motifs show a bimodal distribution with a population of low-scoring and a population of high-scoring motifs. Discordant motifs show a unimodal distribution of low-scoring motifs. B) Proportion of ChIA-PET loops identified by CTCF ChIP-seq peak-based loop prediction at different peak thresholds. The X-axis contains the percentage (x%) of CTCF ChIP-seq peaks used for the prediction, after filtering out those (100-x%) with the lowest scores. C) Length distributions (log10-transformed number of base pairs) of predicted CTCF loops that either overlap (green) or do not overlap (red) experimentally determined Hi-C loops for different cell types. Notches correspond to 95 % confidence intervals for the medians. D) Loop score distributions of predicted CTCF loops that either overlap (green) or do not overlap (red) experimentally determined Hi-C loops for different cell types. Notches correspond to 95 % confidence intervals for the medians. **Figure S3**. Heatmaps showing patterns of predicted CTCF loop-enclosed gene content across different ENCODE cell lines from the University of Washington lab. Genes are clustered using K-means clustering based on their pattern of loop membership across cell lines. Cell lines are hierarchically clustered based on their K-means cluster similarity profiles, using average linkage and Euclidean distance. Heatmap colors indicate the proportion of genes from that cluster falling within CTCF loops in that cell line. A) K-means gene clustering with K = 12 clusters. B) K-means gene clustering with K = 16 clusters. **Figure S4**. Density plot of CTCF motif *p*-values from a genome-wide scan using the FIMO program with a motif *p*-value threshold of 10^−3^. (PDF 326 kb) Additional file 2: Table S1. Gene Ontology term overrepresentation for genes located within and flanking ChIA-PET CTCF loops in K562 and MCF-7 cell lines. (XLSX 11854 kb) Additional file 3: Table S2. Datasets used in and generated by this study. (XLSX 23 kb) Additional file 4: Table S3. Gene Ontology term overrepresentation for genes in different K-means clusters based on occupancy pattern of genes in predicted CTCF loops for the University of Washington (UW) ENCODE cell lines. (XLSX 8869 kb) Additional file 5: A custom Python script. (ZIP 7kb)

## Abbreviations

ChIA-PET: Chromatin interaction analysis by paired-end tag sequencing
ChIP-seq: Chromatin immunoprecipitation followed by high-throughput DNA sequencing
ENCODE: Encyclopedia of DNA elements
H3K4mel: Histone 3 lysine 4 monomethylation
