# Supplementary material for: CTCF-mediated chromatin loops enclose inducible gene regulatory domains
Source: BMC Genomics. 2016 Mar 22;17:252. doi: 10.1186/s12864-016-2516-6 (PMC4804521; doi:10.1186/s12864-016-2516-6)

Supplementary Figure Legends

CTCF-mediated chromatin loops enclose inducible gene regulatory domains

Authors: Martin Oti, Jonas Falck, Martijn A. Huynen, Huiqing Zhou

Supplementary Figure S1: Profiles of histone marks and gene-related features across genomic regions within and around CTCF ChIA-PET loops in K562 cells. The loops and their flanking regions are split into bins each spanning 10% of the loop length. For each bin, the median feature coverage for all loops is plotted. Profiles were normalized by subtracting the mean of all 30 bins, displaying only the variation pattern across the profile. This was done because mean genomic bin coverage can vary substantially between chromatin marks and other genomic features, separating the profiles along the Y-axis and making pattern comparison more difficult. **A)** Loops shorter than 200kb in length. **B)** Loops between 200kb and 1Mb in length. (A & B) Top panels: regulatory element-associated histone marks and variants. Middle panels: Repression-associated histone marks. Bottom panels: Transcribed region-associated histone marks. **C)** Gene-related features for K562 (top) and MCF-7 (bottom) cell lines. (Note that as median densities are plotted per bin, when the proportion of loops with a given feature drops below half the median jumps to zero as in the peripheral bins of the MCF-7 DHS profile. Also note that the strong DHS enrichment at loop anchors compresses the scale of the other profiles.)

Supplementary Figure S2: A) Motif score distributions for exclusively concordant, exclusively discordant and bi-directional loop anchors in both MCF-7 and K562 cells. Concordant and bi-directional motifs show a bimodal distribution with a population of low-scoring and a population of high-scoring motifs. Discordant motifs show a unimodal distribution of low-scoring motifs. **B)** Proportion of ChIA-PET loops identified by CTCF ChIP-seq peak-based loop prediction at different peak thresholds. The X-axis contains the percentage (x%) of CTCF ChIP-seq peaks used for the prediction, after filtering out those (100-x%) with the lowest scores. **C)** Length distributions (log10-transformed number of base pairs) of predicted CTCF loops that either overlap (green) or do not overlap (red) experimentally determined Hi-C loops for different cell types. Notches correspond to 95% confidence intervals for the medians. **D)** Loop score distributions of predicted CTCF loops that either overlap (green) or do not overlap (red) experimentally determined Hi-C loops for different cell types. Notches correspond to 95% confidence intervals for the medians.

Supplementary Figure S3: Heatmaps showing patterns of predicted CTCF loop-enclosed gene content across different ENCODE cell lines from the University of Washington lab. Genes are clustered using K-means clustering based on their pattern of loop membership across cell lines. Cell lines are hierarchically clustered based on their K-means cluster similarity profiles, using average linkage and Euclidean distance. Heatmap colors indicate the proportion of genes from that cluster falling within CTCF loops in that cell line. **A)** K-means gene clustering with K = 12 clusters. **B)** K-means gene clustering with K = 16 clusters.

Supplementary Figure S4: Density plot of CTCF motif p-values from a genome-wide scan using the FIMO program with a motif p-value threshold of 10^{-3}.

Supplementary Figure S1

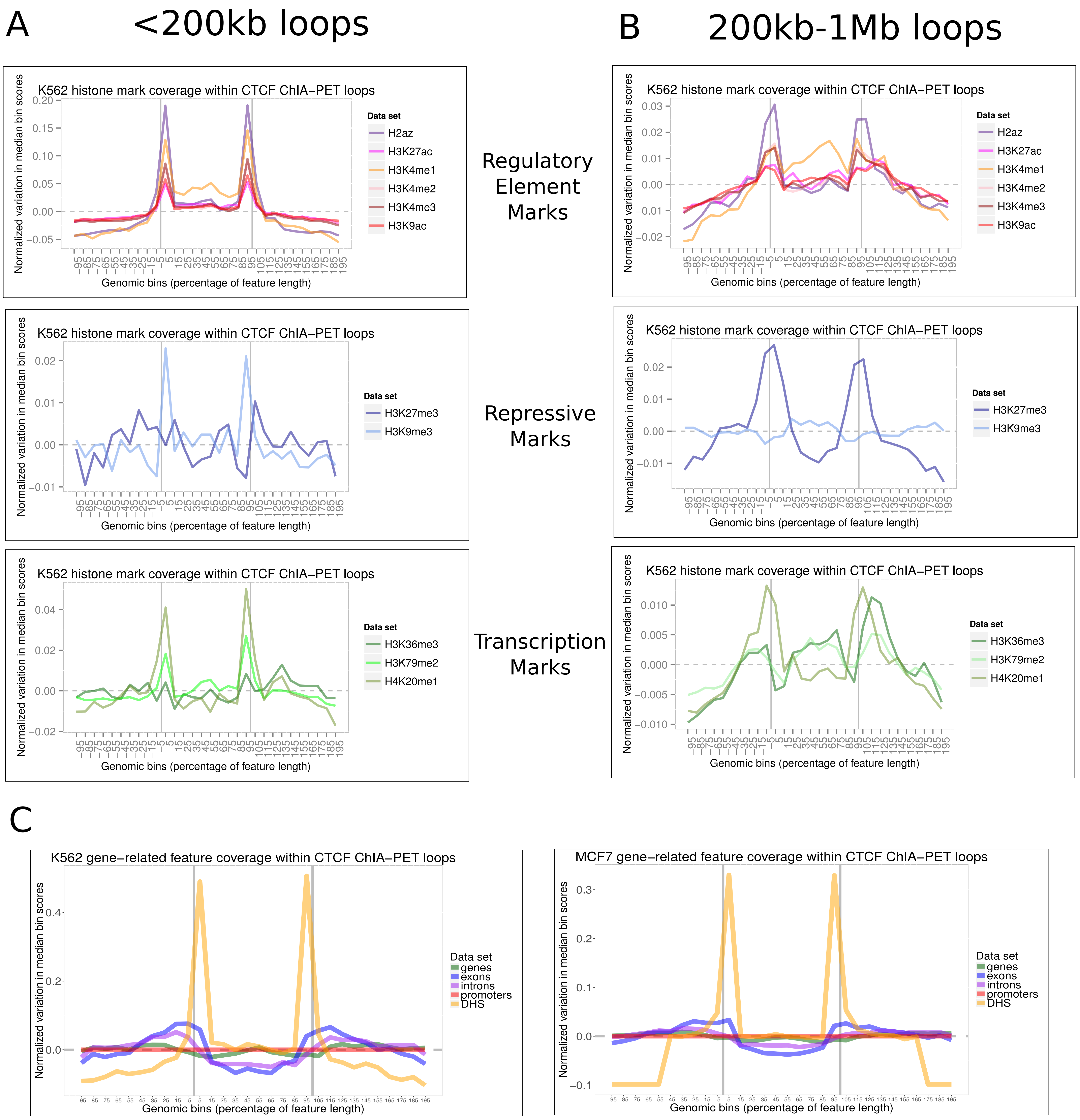

Supplementary Figure S2

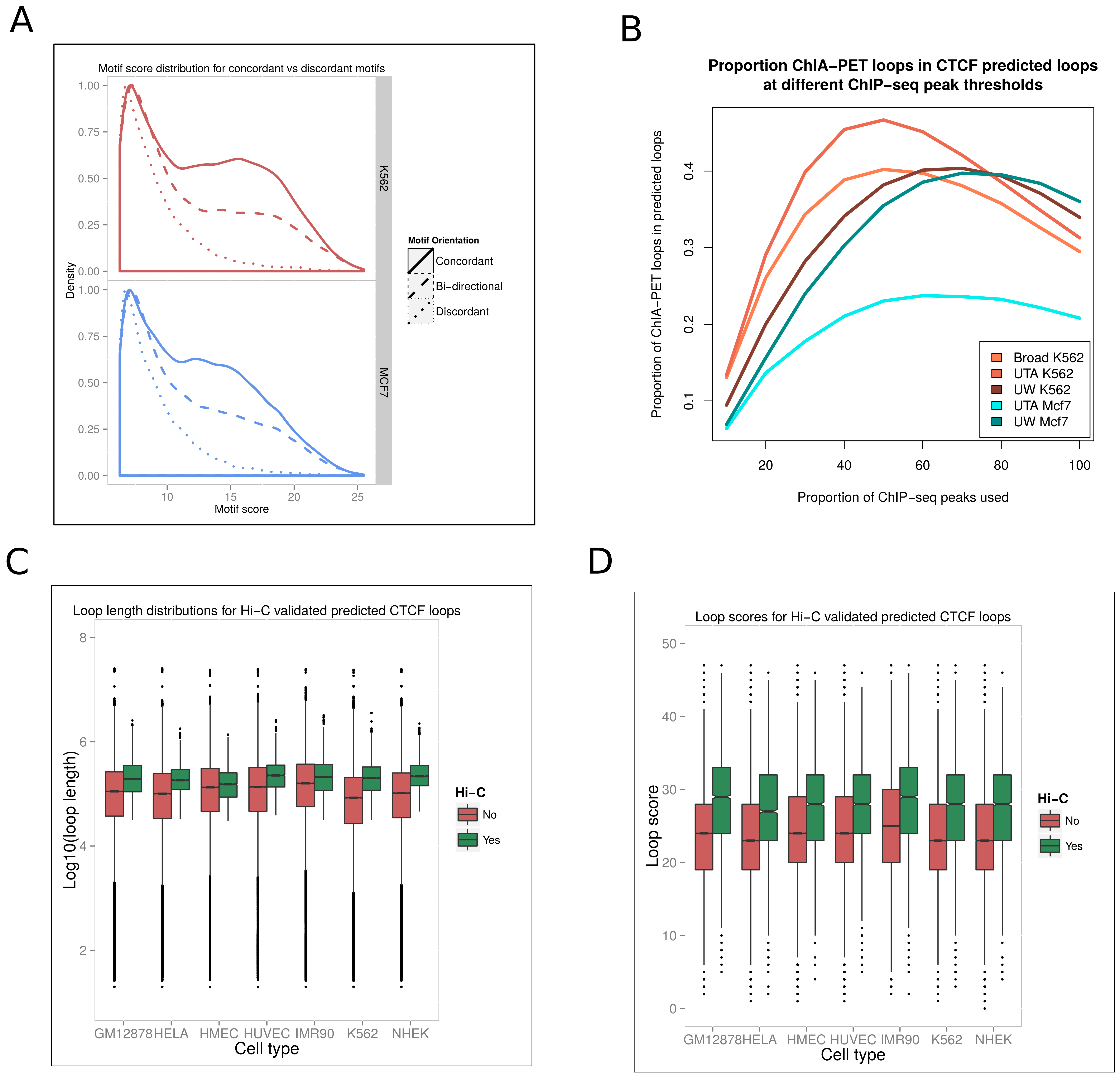

Supplementary Figure S3

Clustering of predicted ENCODE cell line CTCF loop-enclosed genes

A

K = 12 clusters

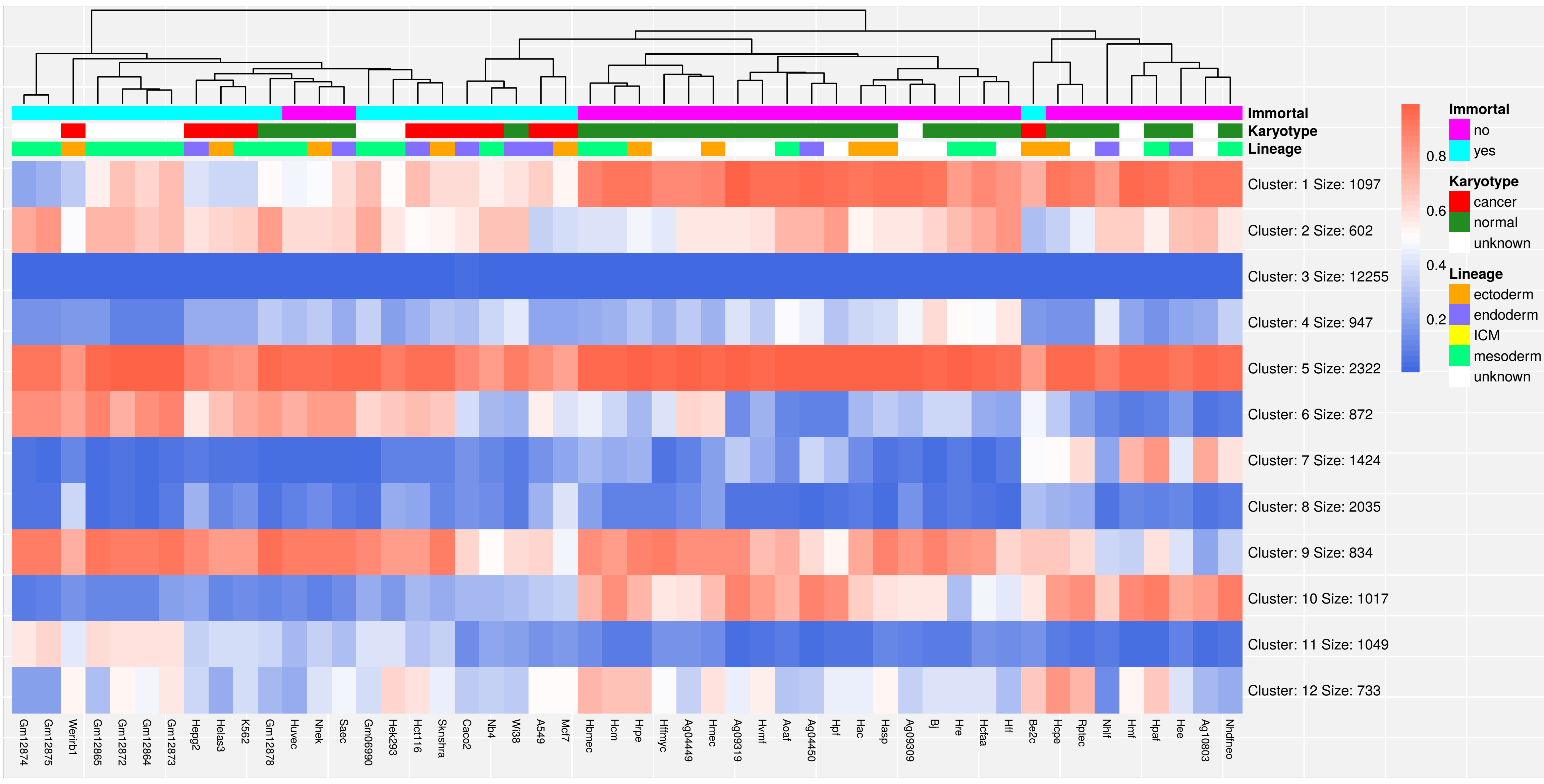

B

K = 16 clusters

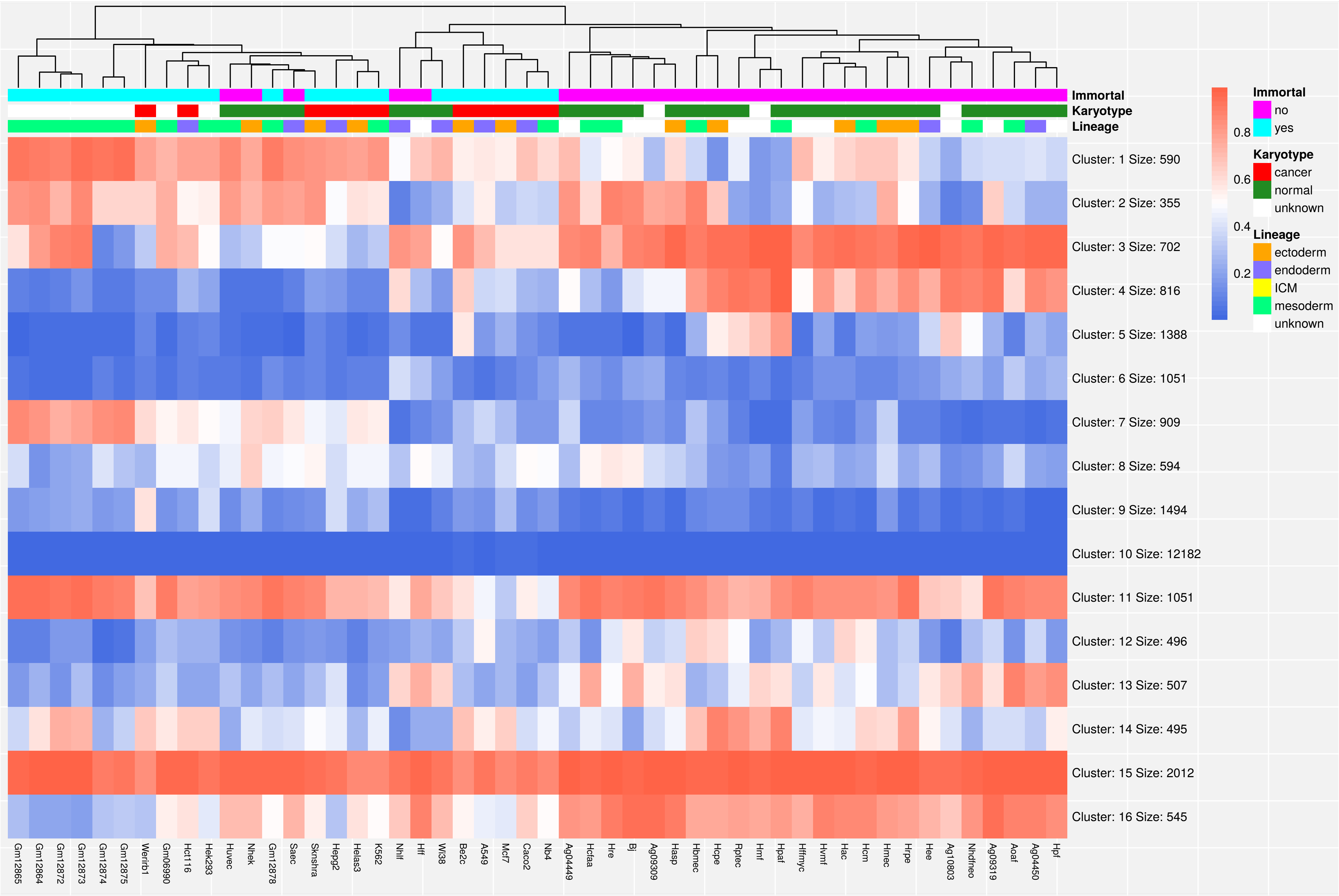

Supplementary Figure S4

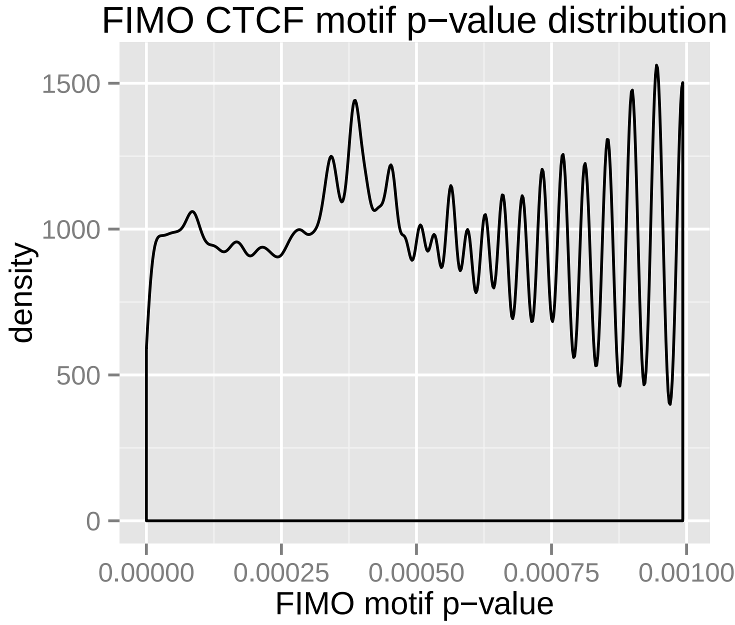

Supplement: Additional file 1: Figure S1. — Profiles of histone marks and gene-related features across genomic regions within and around CTCF ChIA-PET loops in K562 cells. The loops and their flanking regions are split into bins each spanning 10 % of the loop length. For each bin, the median feature coverage for all loops is plotted. Profiles were normalized by subtracting the mean of all 30 bins, displaying only the variation pattern across the profile. This was done because mean genomic bin coverage can vary substantially between chromatin marks and other genomic features, separating the profiles along the Y-axis and making pattern comparison more difficult. A) Loops shorter than 200kb in length. B) Loops between 200kb and 1Mb in length. (A & B) Top panels: regulatory element-associated histone marks and variants. Middle panels: Repression-associated histone marks. Bottom panels: Transcribed region-associated histone marks. C) Gene-related features for K562 (top) and MCF-7 (bottom) cell lines. (Note that as median densities are plotted per bin, when the proportion of loops with a given feature drops below half the median jumps to zero as in the peripheral bins of the MCF-7 DHS profile. Also note that the strong DHS enrichment at loop anchors compresses the scale of the other profiles.). Figure S2. A) Motif score distributions for exclusively concordant, exclusively discordant and bi-directional loop anchors in both MCF-7 and K562 cells. Concordant and bi-directional motifs show a bimodal distribution with a population of low-scoring and a population of high-scoring motifs. Discordant motifs show a unimodal distribution of low-scoring motifs. B) Proportion of ChIA-PET loops identified by CTCF ChIP-seq peak-based loop prediction at different peak thresholds. The X-axis contains the percentage (x%) of CTCF ChIP-seq peaks used for the prediction, after filtering out those (100-x%) with the lowest scores. C) Length distributions (log10-transformed number of base pairs) of predicted CTCF loops that either overlap [file 12864_2016_2516_MOESM1_ESM.pdf]
